# Effects of Body Weight Support-Tai Chi Footwork Training on Balance Control and Walking Function in Stroke Survivors with Hemiplegia: A Pilot Randomized Controlled Trial

**DOI:** 10.1155/2020/9218078

**Published:** 2020-12-19

**Authors:** Xiao-Ming Yu, Xue-Ming Jin, Yan Lu, Yang Gao, Hai-Chen Xu, Xin Xue, Lei Fang, Jun Hu

**Affiliations:** ^1^Department of Rehabilitation, Shanghai Seventh People's Hospital, Shanghai University of Traditional Chinese Medicine, Shanghai 200137, China; ^2^School of Rehabilitation Medicine, Shanghai University of Traditional Chinese Medicine, Shanghai 201203, China; ^3^Department of Medical Image, Fanxian People's Hospital, Puyang, Henan 457500, China

## Abstract

**Background:**

Tai Chi (TC) is known to enhance balance control and walking function in stroke survivors. However, motor disorders in stroke patients may limit the implementation of TC exercise and increase the risk of falling. The body weight support (BWS) device can provide protection during the early rehabilitation of stroke survivors using an overhead suspension system. Theoretically, combining TC with BWS may be an effective intervention for stroke survivors. This study aimed to examine the effects of body weight support-Tai Chi training on balance control and walking function in stroke survivors with hemiplegia.

**Methods:**

Seventy-one stroke survivors with hemiplegia aged 30–75 years were randomly allocated to the control group (*N* = 35) or the BWS-TC group (*N* = 36). During BWS-TC training, the subjects performed 7 Tai Chi footwork forms, and gradual easy-to-difficult progression (from 40% to 0% body weight) was followed. The subjects participated in 40 min rehabilitation sessions three times per week for 12 weeks. The primary outcome was dynamic balance in the limits-of-stability test. The secondary outcomes, which reflect improvements walking function, included spatiotemporal parameters, the joint range of motion in the affected limb during the swing phase, the Berg Balance Scale score, and the Fugl-Meyer Assessment score. Evaluations were performed at baseline and 12 weeks and compared between groups.

**Results:**

After training, significant between-group differences were observed in the scores for overall, forward, left, right, forward-left, and forward-right directional control in the limits-of-stability test (*P* < 0.05). Furthermore, the scores for gait cycle time, step length, step velocity, and range of motion of the joints were better in the BWS-TC group than in the control group (*P* < 0.05).

**Conclusions:**

The 12-week BWS-TC training may enhance dynamic balance and walking function in stroke survivors with hemiplegia.

## 1. Introduction

Stroke is the second most common cause of death and a leading cause of long-term disability among middle-aged and elderly adults worldwide [1, 2]. An epidemiological study reported that 70–80% of stroke survivors develop functional disabilities [3, 4]. Chronic paralysis and motor control deficits contribute to significant limitations in physical and social functioning and impose a huge public health burden [5]. Exercise intervention is an integral part of the rehabilitation for motor impairments caused by stroke and has been shown to improve the walking function, balance control, and functional independence [6–8]. However, most exercise interventions, such as resistance-based exercises [6], body weight support treadmill training [9], virtual reality [10], and passive robots [11], require safety monitoring and are equipment-dependent. Therefore, the study and development of alternative forms of exercise that could improve motor function of stroke survivors are necessary.

Tai Chi (TC) is an ancient form of exercise that has been applied in stroke rehabilitation for over 10 years worldwide [2, 12]. As a balance-based exercise, TC has been demonstrated to improve strength, balance, and physical function and prevent falls in older adults [13, 14]. Recent system reviews and meta-analyses suggest that it may also improve postural control and balance ability of stroke survivors [2, 15]. However, for those who do participate in TC rehabilitation programs, various exercise-related impairments such as spasticity, weakness, proprioceptive deficit, abnormal agonist-antagonist coactivation, and fear of falling can pose significant barriers to compliance [16, 17]. Consequently, functional gains are not commonly achieved, leading most stroke patients to feel discouraged and to discontinue treatment [18].

Body weight support (BWS) treadmill training has shown promise in providing improvements in motor function, locomotion ability, and balance in stroke survivors [19]. The BWS treadmill system consists of an over suspension system (i.e., BWS) and a treadmill. A certain percentage of the subject's body weight is supported by the overhead suspension system via a harness worn by the subject during walking [20]. BWS can provide stroke survivors with confidence in starting rehabilitation early after surgery or trauma to regain balance and locomotion without the fear of falling [21]. In addition, BWS reduces lower extremity load, thus facilitating step initiation [22]. However, BWS treadmill training is a fixed system that can only be used for gait training and cannot be combined with other exercise interventions. Additionally, BWS gait training focuses on the improvement of function in the sagittal plane, while TC requires the subject to execute symmetric and diagonal movements, controlling the center of gravity around and over the edge of the base of support [23]. TC and BWS have unique advantages and their individual application in rehabilitation has been confirmed to provide positive effects for stroke survivors. Theoretically, combining TC with BWS may be an effective intervention for improving balance control and gait function in stroke survivors.

Therefore, we propose a novel intervention using combined TC and BWS and herein aimed to examine the effects of BWS-TC training on balance control and gait function in stroke survivors with hemiplegia. Because this exercise program emphasized weight shifting in different footwork and lower extremity control movements near the limits of stability (LOS) [24], we hypothesized that the BWS-TC footwork training could improve balance control. In addition, a previous study reported that BWS decreases the lower extremity load and facilitates step initiation [22]. Therefore, we further hypothesized that the BWS-TC footwork training could improve lower limb function and gait spatiotemporal pattern.

## 2. Materials and Methods

### 2.1. Study Design and Participants

**Figure 1:**
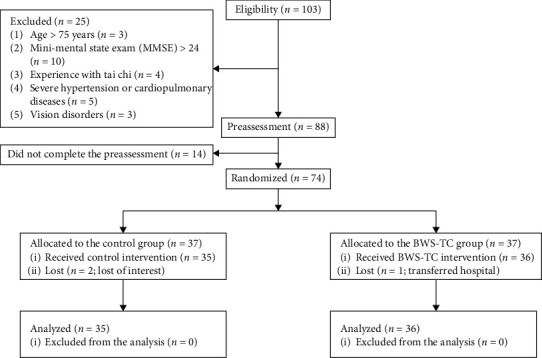
Flowchart illustrating the process of subject recruitment and retention. MMSE: mini-mental state examination; BWS-TC: body weight support-Tai Chi.

This assessor-blinded randomized controlled clinical trial included stroke patients recruited from the Shanghai Seventh People's Hospital and community centers in the vicinity (Gaoqiao, Pudong District, Shanghai, China) using flyers, posters, and referrals from neurologists and physical therapists between March 2019 and November 2019. The inclusion criteria were as follows [25–27]: clinical diagnosis of cerebral hemorrhage or infarction by computed tomography/magnetic resonance imaging, aged 30–75 years, ≥3 months since stroke onset, a score of >24 on the Mini-Mental State Examination, able to stand unaided and walk without an assistive device, and no prior experience of TC. The exclusion criteria included current involvement in any other clinical study or instructor-directed exercise program, vision disorders, severe hypertension or cardiopulmonary diseases, and lower extremity joint or muscle injuries [27]. The flowchart of subject recruitment and retention is shown in Figure 1. This study was approved by the institutional review board of Shanghai Seventh People's Hospital (2018-IRBQYYS-012). Informed consent was obtained from all participants enrolled in the study. The trial was registered with the ClinicalTrials.gov (ChiCTR1900020758).

### 2.2. Sample Size

The sample size was calculated using G*∗*power software (v3.1.9.2, University Dusseldorf, Germany; available for download from http://www.psychologie.hhu.de/arbeitsgruppen/allgemeine-psychologie-und-arbeitspsychologie/gpower.html) based on a comparison of outcome measures between the BWS-TC and control groups, represented by improvement in dynamic balance in the LOS as the primary outcomes. During the preliminary study, we randomly assigned 26 subjects with stroke to the control group and the BWS-TC group. The subjects participated in 40 min rehabilitation sessions three times per week for 4 weeks. Our preliminary test data indicated that the means ± standard deviations of the scores were 9.62 ± 4.16 points and 5.05 ± 4.62 points in the BWS-TC and control groups, respectively. According to a prior two-way analysis of variance (ANOVA) *F*-test, with a power of 0.80, an alpha level of 0.05, and a fall rate of 20%, an estimated 40 participants were required for this study.

### 2.3. Randomization and Allocation Concealment

The participants were screened through in-person evaluation to determine if they met the inclusion and exclusion criteria. After completing baseline testing, each participant received a sealed envelope containing a random allocation sequence number to either the BWS-TC group or control group. The sequence numbers were generated by an independent statistician using Excel (Microsoft, USA). The statistician, outcome assessors, and data analyzers were blinded to study recruitment, intervention, and evaluation.

### 2.4. Interventions

The BWS-TC group received a combination of BWS-TC footwork training and conventional rehabilitation therapies, while the control group received conventional rehabilitation therapies. Ten junior level physical therapists with >5 years of clinical experience performed routine physical therapy and TC intervention. The subjects participated in 40 min rehabilitation sessions three times per week for 12 weeks.

#### 2.4.1. BWS-TC Group

**Figure 2:**
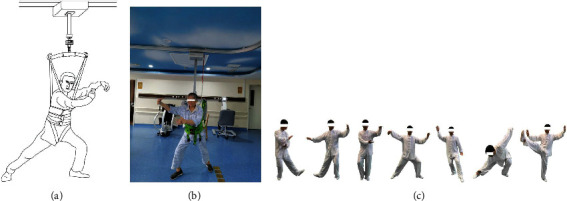
Schematic diagram of body weight support-Tai Chi (BWS-TC) footwork training. (a) Sketch map of BWS-TC. (b) Example diagram of BWS-TC. (c) Seven step forms of Tai Chi footwork (forward steps; backward steps; shuffle steps; lunge steps; empty steps; turn around; single-leg support).

The TC intervention applied in this study was chiefly based on BWS training. As shown in Figure 2, each patient was asked to wear a harness, and a specific percentage of their body weight was supported by an overhead suspension system (LiKorallTM, 250 ES, Hill-Rom, Sweden). The TC intervention was developed based on the 24-form simplified TC promoted by the State Sports General Administration of China [28]. From the 24 forms of the simplified TC, we selected seven step forms: forward steps, backward steps, shuffle steps, empty steps, lunge steps, single-leg support, and turning around (Figure 2). The footwork is the foundation and precursor of TC exercise [29], and these seven typical step forms comprise most TC movements. The BWS-TC footwork training program was designed to minimize the effect of motor impairment for TC rehabilitation. We aimed to improve their insufficient support capacity to preserve motor function as well as balance control. This was achieved by asking the subjects to implement both symmetric and diagonal movements, including weight shifts, controlled displacement of the center of gravity over their base of support, ankle sways, and anterior-posterior and lateral stepping. Therefore, this training did not focus on the movements of the upper limbs and mainly emphasized endurance among different movements as well as weight shifts.

In the present study, two martial art coaches were hired to teach TC footwork. These two coaches had national second-level athlete certifications from the National Traditional Sports Major of Shanghai University of Sports. Previous studies reported a significant reduction in energy cost and quadriceps activation at a BWS of 40% [19, 30]. As the level of BWS increased, lower limb and muscle activity progressively decreased. Therefore, the initial BWS was set as at 40% in the current study. During the 12-week BWS-TC program, a gradual easy-to-difficult progression was followed, which was divided into five stages corresponding to BWS: week 1, 40%; weeks 2-3, 30%; weeks 4–6, 20%; weeks 7–9, 10%; and weeks 10–12, 0%. The BWS-TC group was required to undergo a 40 min session (20 min conventional rehabilitation programs and 20 min TC) three times per week for 12 weeks.

#### 2.4.2. Control Group

The control group received conventional rehabilitation programs, including active mobilization of the limb muscles and joints, proprioceptive neuromuscular facilitation, muscle resistance training, stretching training, sit-to-stand training, and walking. The control group was required to undergo a 40 min rehabilitation session three times per week for 12 weeks.

### 2.5. Outcome Measures

All evaluations were performed before and 12 weeks after intervention by a rehabilitation assessor who was not part of the clinical study team. The primary outcome was dynamic balance in LOS, as measured by computerized dynamic posturography (Biodex Balance System, USA). The secondary outcomes included spatiotemporal parameters; the range of motion (ROM) of the ankle, knee, and hip during the affected limb swing phase of the gait cycle; Berg Balance Scale (BBS) score; and Fugl-Meyer Assessment (FMA) score of the lower limbs. Thus, the subjects were evaluated on five outcome measures at baseline and 12 weeks. A single evaluator with significant assessment experience performed the evaluations to eliminate variability in assessment results and to ensure assessment accuracy.

#### 2.5.1. Primary Outcomes

The LOS was defined as the farthest distance in eight directions where a subject could lean from an upright position within their base of support without taking any steps [24]. A number of studies have demonstrated that the LOS test is a sensitive measure of dynamic balance control, and the reliability of evidence shows moderate test-retest reliability for directional control [31]. In this study, the LOS test was set to the easy level (50%) due to the physical impairment of the stroke survivors. All BBS procedures were performed according to the manufacturer's guidelines. The LOS score ranged from 0 to 100%, with higher scores indicating better balance control. Only one successful trial was collected to disregard learning effects and avoid muscle tiredness.

#### 2.5.2. Secondary Outcomes

*(1) Gait Analysis*. The ODONATE gait analysis system (ODONATE, Maver, Shanghai, China) was used to evaluate the temporospatial parameters and ROM of the lower limb joints in the sagittal plane during walking.

The ODONATE gait analysis system was used to collect the point cloud on the human body surface and to automatically analyze the walking performance. In the supplementary attachments (available here), we submitted the comparative results of gait biomechanical between ODONATE and the gold standard in the motion capture system (Vicon Motion Systems, Oxford, United Kingdom). Furthermore, the interclasses correlation coefficient (ICC) results indicated that the ODONATE system has high reliability and validity in gait analysis (ICC Hip: 0.990; Knee: 0.997; Ankle: 0.982; see the Supplementary Materials).

Previous studies demonstrated that a self-selected walking speed is a good indicator of overall gait performance and is commonly used to assess locomotor ability [32]. Thus, subjects were instructed to walk at a self-selected speed on a 10 m walkway, and the temporospatial parameters and angle of the lower limb joints in the sagittal plane were collected. The gait cycle was defined as the period beginning with the unaffected limb's heel contact to its next heel contact [33]. Step length was measured from heel to heel in the anterior-posterior direction. The affected limb single support time (SST) was calculated from the unaffected limb toe-off to the ipsilateral heel contact [33]. The double support time (DST) was measured as follows: the first from the heel contact of the unaffected limb heel to the toe-off of the affected limb and the second from the heel contact of the affected limb to the toe-off of the unaffected limb. Furthermore, the ROM of the ankle, knee, and hip during the affected limb swing phase of the gait cycle was determined [34]. Each variable from the three trials for each subject was then averaged for subsequent statistical analysis.

*(2) Lower Limb Motor Function Assessment*. The motor function of lower limbs was measured by simplified Fugl-Meyer Assessment (FMA) scale, which has demonstrated excellent interrater and intrarater reliability and construct validity and is often used in stroke rehabilitation research [35, 36]. The aggregate score of scale is 34, with higher scores indicating less motor damage.

*(3) Berg Balance Assessment*. The Berg Balance Scale (BBS) was used to measure the balance score of the subjects in this study. Recent systematic review reported that the BBS has high intra- and interrater relative reliability in balance assessment for the poststroke population [37]. Each of these items is scored from 0 to 4, which are summed to make a total score between 0 and 56, with a higher score indicating better balance.

### 2.6. Statistical Analysis

All statistical analyses were performed using IBM SPSS version 20.0 (SPSS Inc., Chicago, IL, USA). The primary and secondary outcomes analyses were performed on an intention-to-treat basis. Between-group differences in demographic and baseline variables were tested using the chi-square test for categorical variables and a one-way ANOVA for continuous variables. A two-way repeated measures ANOVA with group as a group factor and time factor was used to calculate the effects of the interventions on all outcome measures. Simple effect analysis was conducted using SYNTAX grammar, where the time × group interaction effect was significant. An alpha level of 0.05 was considered as statistically significant.

## 3. Results

### 3.1. Baseline Characteristics of the Subjects

The demographic and baseline characteristics of the 71 subjects are presented in Table 1. There were no significant differences between the two groups (*P* > 0.05). During the study period, no adverse events were observed.

### 3.2. Primary Outcomes

Table 2 presents the between-group differences in dynamic control at baseline and 12 weeks. Significant group × time interactions were observed in complete time, right, forward-left, and forward-right directional control during the LOS test (*P* < 0.05). There was no significant preintervention difference between the two groups, as per the simple effect result of the group factor (*P* > 0.05). However, significant group differences were observed in complete time, right, and forward-left directional control during postintervention (*P* < 0.05). Furthermore, we also observed significant between-group changes in the scores for overall, forward, left, right, forward-left, and forward-right directional control (*P* < 0.05). Additionally, we found a significant time effect in the score of overall, right, forward-left, forward-right, and backward-right directional control and complete time between the two groups (*P* < 0.05).

### 3.3. Secondary Outcomes

Table 3 shows the comparison of spatiotemporal parameters and joint ROM of the affected limb during gait between the two groups at baseline and 12 weeks. We observed significant group × time interactions in the gait cycle time, step length, and hip swing range between the two groups (*P* < 0.05). The simple effect results showed that the group differences in the gait cycle time, step length, and hip swing range were statistical significant during post-intervention (*P* < 0.05). In addition to the SST and DST, significant group and time differences were observed between the two groups in other spatiotemporal parameters and the joint ROM of the affected limb during walking (*P* < 0.05).

Table 4 shows the comparisons of FMA and BBS scores between the two groups at baseline and 12 weeks. Significant interactions were found in the FMA and BBS between the two groups. We observed the significant differences in both indices during postintervention between the two groups, as per the simple effect result of the group factor (*F* = 4.764, *P*=0.046; *F* = 15.213, *P*=0.000). Additionally, the main effect of group in BBS was significant between the two groups (*F* = 6.561, *P*=0.039). Although the two groups exhibited a mean increase of 56.77% and 34.02% between baseline and 12 weeks, respectively, no significant group difference was found.

## 4. Discussion

Stroke survivors do not commonly achieve functional gains in the traditional TC rehabilitation programs due to the presence of various motor-related impairments [17]. In the present study, we proposed a novel intervention strategy that combined TC and BWS and examined the effects of BWS-TC training on balance control and walking function in stroke survivors with hemiplegia. As hypothesized, the BWS-TC footwork training program improved balance control and walking function. Furthermore, no adverse events were observed during the study period. These results indicate the safety and utility of this combined intervention for improving balance control and walking function in stroke survivors with hemiplegia.

Balance control for the dual purposes of maintaining postural stability and orientation is critical to the functional performance of activities of daily living and preventing falls [13, 24]. In the present study, we observed significant between-group differences in overall, forward, left, right, forward-left, and forward-right directional control of dynamic balance. Although the magnitude of change in the other indices in the BWS-TC group was greater than that in the control group, the between-group differences were not significant. These results suggest that 12-week BWS-TC footwork training was beneficial and effective in improving dynamic balance in stroke survivors. Previous studies have also reported similar findings [4, 13, 27]. In addition, the BBS score, which reflects balance, was significantly improved in the BWS-TC group compared to the control group. However, the beneficial rehabilitation effects of TC were not consistent in previous studies. Takeshima et al. [38] reported that 12 weeks of TC exercise had no significant effect on balance and functional fitness parameters in older Japanese adults (average age, 72 years). One of the reasons for the lack of improvement may be that TC is complex and difficult for older Japanese adults who are not accustomed to this activity to perform. During TC training without protection, the degree of functional improvement and increased incidence of adverse events, such as falling, are interdependent [39]. Therefore, another possible explanation for no improvement might be that the subjects selected a moderate or lower TC exercise intensity (e.g., higher center of gravity), based on their functional level, fear of falling, and risk control.

From the perspective of exercise training, a moderate or nonstimulating exercise load may have no significant effect on improving muscle strength and balance control [40]. However, the incidence of adverse events will increase during training when the focus is on the pursuit of functional enhancement. Adverse event reporting within clinical trials is an important source for evaluating the safety of new therapies [39]. A number of TC studies have provided evidence of its clinical efficacy and cost effectiveness, particularly among older adults and those deconditioned by chronic illness, but some adverse events have also been reported [24, 41, 42]. In the present study, no adverse events were observed. Thus, the significant positive rehabilitation effects on balance control and lack of adverse events observed herein may be related to simple footwork training under the protection of BWS, which can gradually improve function with the challenge of the LOS without fear of falling.

The recovery of walking function is the primary focus of stroke rehabilitation [3, 43]. As functional measures of walking, we used the gait spatiotemporal variables and lower limb joint ROM referring to the previous literature [34]. Our results revealed that the improvements in the cycle time, step velocity, step length, and lower limb joint ROM were greater in the BWS-TC group than in the control group. Similarly, Yang [44] reported significant differences in measures such as step length (from 0.33 to 0.42 m) and walking speed (from 0.44 to 0.57 m/s) after a 4-week modified TC intervention. A number of previous studies also observed improvements in walking speed on the 10 m walking test and timed-up-and-go after TC intervention [45]. Zou et al. [46] demonstrated that the practice of TC has a positive effect in improving joint ROM in healthy older women. Although our findings are similar to those of previous studies, it is difficult to discuss the additional gains in BWS via comparison with previous studies due to differences in the baseline characteristics, duration of intervention, and outcome measures in the above studies. In future research, TC training without BWS should be included as an additional control intervention.

The current study demonstrated that BWS-TC footwork training can improve balance control and walking function in stroke survivors with hemiplegia. Furthermore, our lower extremity function results revealed significant differences between the two groups. This partially explains the possible reasons for the enhanced balance control and walking function of stroke survivors in the BWS-TC group. Such significant positive rehabilitation effects may be related to the unique rehabilitation program. BWS-TC footwork training is practiced on each side to improve movement coordination and symmetry through repetitive bilateral and reciprocal limb movements. The program translates the dualities into a dynamic exchange of stability (movements within the base of support) and instability (movements on the periphery of the base of support). As such, training involves voluntarily controlled TC postural movement excursions of the center of gravity over and/or around the edge of the base of support, with the goal of increasing the sway envelope and thereby expanding the LOS [23]. In addition, the presence of BWS makes it easier in footwork self-initiated and control movement for stroke patients to create postural sway at the ankle and/or hip to engage participants in adaptive training of balance control [20]. More importantly, external protection devices provide a basis for stroke survivors to perform various types of training without fear of falling [29]. The enhancement of the ROM and control of the ankle and hip joints not only were conducive to balance control, but also had a positive effect on improving gait and walking function. These results indicate the safety and utility of this combined intervention strategy in improving balance control and walking function in stroke survivors with hemiplegia and provide insight into the design of rehabilitation interventions for fall prevention.

This study also has some limitations. First, we only used routine rehabilitation treatment as a control and did not investigate any other types of exercise interventions. Second, a previous study has recommended that stroke patients self-practice either with their family or in their community once the interventions are complete [21, 47]. However, due to the lack of BWS equipment in these locations, we did not conduct any follow-up after the interventions were completed. Third, the synergistic movement and spasticity level of lower limb muscles of subjects were not assessed. However, during the intervention, no apparent synergistic movement or spasticity of the lower limb muscles was observed. This may be related to the longer time after onset (average 11.36 months in the BWS-TC group and 9.38 months in the control group) and reduced load and requirement of lower limb muscle activation due to BWS. Finally, the proprioceptive input and sensory integration system may play an important role in balance control and walking function [27]. However, we did not assess the change in sensory integration. Nevertheless, our study suggests that BWS-TC footwork training is useful for improving dynamic balance and walking function in stroke survivors.

## 5. Conclusion

Twelve weeks of body weight support-Tai Chi footwork training improved dynamic balance control and walking function of stroke survivors with hemiplegia. The future work should be to set Tai Chi training without body weight support as the control and to explore the effect of body weight support in function improvement.

## Figures and Tables

**Table 1:** Demographic and clinical characteristics of the study subjects at baseline.

Characteristic	BWS-TC (*n* = 35)	Control (*n* = 36)	*F*/*χ*^2^	*P*
Age (years)	63.03 ± 8.92	58.69 ± 9.72	3.872	0.054
Body mass (kg)	67.81 ± 8.66	65.06 ± 8.15	1.976	0.164
Time after onset (months)	11.38 ± 5.21	9.41 ± 4.82	2.241	0.143
MMSE (score)	27.52 ± 1.88	28.19 ± 2.04	1.743	0.191
Sex (male/female)	21/14	20/16	0.705	0.445
Hemiparesis side (left/right)	18/17	21/15	0.559	0.365
Stroke type (Isc/Hem)	25/10	22/14	0.358	0.252

Continuous variables are presented as mean ± SD; BWS-TC: body weight support-Tai Chi; MMSE: mini-mental state examination; Isc: Ischemic; Hem: hemorrhagic.

**Table 2:** Comparison of dynamic control between two groups.

Index	Preassessment		Postassessment		Group × time (*F*/*P*)	Group (*F*/*P*)	Time (*F*/*P*)	Simple effect (*F*/*P*)	
	BWS-TC	Control	BWS-TC	Control				Pre	Post
Overall	29.94 ± 7.87	27.71 ± 9.92	46.54 ± 14.12	33.14 ± 12.02	N.S.	8.365/0.009	6.726/0.024	N.S.	N.S.
Complete time	65.74 ± 13.94	64.67 ± 11.18	45.06 ± 10.3	49.07 ± 7.89	6.612/0.024	N.S.	14.234/0.000	N.S.	4.215/0.046
Forward	40.87 ± 7.01	36.98 ± 10.89	57.36 ± 8.63	44.98 ± 11.78	N.S.	5.368/0.041	N.S.	N.S.	N.S.
Backward	18.15 ± 6.34	16.65 ± 6.22	27.57 ± 9.37	19.76 ± 6.28	N.S.	N.S.	N.S.	N.S.	N.S.
Left	37.04 ± 8.86	37.78 ± 15.51	53.82 ± 7.32	40.10 ± 14.08	N.S.	9.951/0.008	N.S.	N.S.	N.S.
Right	36.14 ± 12.99	37.41 ± 19.62	58.21 ± 8.60	41.24 ± 12.54	4.566/0.048	12.324/0.000	5.212/0.038	N.S.	12.258/0.000
Forward-left	42.12 ± 10.65	45.92 ± 9.56	54.93 ± 6.05	49.37 ± 16.41	6.235/0.027	5.522/0.037	4.967/0.047	N.S.	4.962/0.044
Forward-right	45.66 ± 9.61	46.43 ± 7.87	56.23 ± 11.34	52.49 ± 14.76	5.623/0.033	4.868/0.044	4.268/0.049	N.S.	N.S.
Backward-left	21.15 ± 7.10	19.27 ± 7.34	26.12 ± 6.12	25.61 ± 10.23	N.S.	N.S.	N.S.	N.S.	N.S.
Backward-right	23.41 ± 8.43	22.42 ± 10.32	34.54 ± 11.56	26.12 ± 6.12	N.S.	N.S.	5.298/0.034	N.S.	N.S.

BWS-TC: body weight support-Tai Chi; N.S.: no significant difference.

**Table 3:** Comparison of spatiotemporal parameters and joint range of affected limb during gait between two groups.

Index	Preassessment		Postassessment		Group × time (*F*/*P*)	Group (*F*/*P*)	Time (*F*/*P*)	Simple effect (*F*/*P*)	
	BWS-TC	Control	BWS-TC	Control				Pre	Post
Gait cycle time (s)	1.38 ± 0.24	1.31 ± 0.26	1.13 ± 0.19	1.18 ± 0.24	8.968/0.011	4.996/0.041	10.268/0.004	N.S.	4.024/0.047
Step velocity (m/s)	0.45 ± 0.11	0.42 ± 0.12	0.67 ± 0.18	0.51 ± 0.21	N.S.	9.62 2/0.007	8.695/0.010	N.S.	N.S.
Step length (m)	0.25 ± 0.14	0.28 ± 0.12	0.38 ± 0.15	0.32 ± 0.11	13.368/0.000	4.368/0.049	7.102/0.014	N.S.	5.196/0.034
SST_AL_ (%)	33.68 ± 9.62	31.36 ± 10.23	39.26 ± 11.23	35.77 ± 10.96	N.S.	N.S.	N.S.	N.S.	N.S.
DST (%)	26.16 ± 15.42	27.29 ± 14.27	22.12 ± 14.39	24.36 ± 13.83	N.S.	N.S.	N.S.	N.S.	N.S.
Hip swing range (°)	37.26 ± 11.23	38.52 ± 12.45	47.87 ± 12.88	43.74 ± 11.57	15.336/0.000	4.522/0.047	9.217/0.010	N.S.	5.961/0.032
Knee swing range (°)	32.21 ± 13.25	31.85 ± 11.98	45.33 ± 14.26	39.55 ± 12.03	N.S.	6.462/0.021	12.011/0.000	N.S.	N.S.
Ankle range (°)	13.22 ± 4.26	12.38 ± 5.12	21.64 ± 5.36	19.98 ± 7.62	N.S.	7.987/0.015	14.368/0.000	N.S.	N.S.

BWS-TC: body weight support-Tai Chi; N.S.: no significant difference; AL: affected limb; SST: single support time; DST: double support time.

**Table 4:** Comparison of FMA and BBS between the two groups.

Index	Group	Pre	Post	Change (%)
FMA^*∗*^^*‡*#^	BWS-TC	15.91 ± 6.54	25.17 ± 5.35	56.77
	Control	16.17 ± 5.92	21.78 ± 7.83	34.02
BBS^*∗*^^*‡*#*§*^	BWS-TC	33.40 ± 8.83	48.03 ± 9.59	44.62
	Control	34.50 ± 8.41	39.64 ± 12.39	14.51

FMA: fugl-meyer assessment; BBS: berg balance Scale; BWS-TC: body weight support-Tai Chi; Pre: preassessment; Post: postassessment. ^*∗*^Statistically significant interaction between group and time. ^*‡*^Statistically significant group difference in the postassessment based on simple effect test. ^#^Statistically significant time difference. ^*§*^Statistically significant group difference.

## Data Availability

The raw data supporting the conclusions of this article will be made available by the authors, without undue reservation, to any qualified researcher.

## Supplementary Materials

Supplementary Materials The comparative results of gait biomechanical between ODONATE and the gold standard in the motion capture system (Vicon Motion Systems, Oxford, United Kingdom) are provided. Furthermore, the interclasses correlation coefficient (ICC) results indicated that the ODONATE system has high reliability and validity in gait analysis (ICC Hip: 0.990; Knee: 0.997; Ankle: 0.982).
