# Supplementary material for: Effects of Body Weight Support-Tai Chi Footwork Training on Balance Control and Walking Function in Stroke Survivors with Hemiplegia: A Pilot Randomized Controlled Trial
Source: Evid Based Complement Alternat Med. 2020 Dec 19;2020:9218078. doi: 10.1155/2020/9218078 (PMC7769631; doi:10.1155/2020/9218078)
Supplement: Supplementary Materials — The comparative results of gait biomechanical between ODONATE and the gold standard in the motion capture system (Vicon Motion Systems, Oxford, United Kingdom) are provided. Furthermore, the interclasses correlation coefficient (ICC) results indicated that the ODONATE system has high reliability and validity in gait analysis (ICC Hip: 0.990; Knee: 0.997; Ankle: 0.982). [file 9218078.f1.pdf]

Methods:

Test A： Several experimenters conducted a set of natural walking experiments on the same subjects.

Test instructions:

experimenter	Including 5 people been trained at the same time and have not participated in the main experiment.
subject	Age 27, height 176cm, weight 72kg, no gait-disorder related diseases.
A group of natural walking test.	The subjects walked normally 5 times effectively.
Effective normal walking	The subject maintains a natural state, and the walking distance includes more than 3 complete gait cycles.

Statistic calculation:

(1) A complete gait cycle collected in the natural walking test is normalized to 100%, and 0% is heel strike.

(2) The data of all gait periods obtained from 5 effective normal walks in a group of natural walking test were taken to obtain an average gait period (Select the left leg data uniformly).

(3) Calculate the intraclass correlation coefficient (ICC) of the average gait cycle data obtained by all different experimenters to evaluate the reliability of repeated measures.

(4) Calculate the range of motion (ROM) of lower joints on each plane in the mean gait cycle.

(5) Calculate the relative standard deviation (RSD) of the angular mobility obtained by all experimenters in the walking test, and evaluate the precision of the experiments results.

(6) Calculate the RSD of the spatiotemporal parameters of the walking test obtained by all experimenters.

Test B: An experimenter repeatedly measured the natural walking experiments of a subject several repeated times.

experimenter	The people participating in this experiment.
subject	Age 30，height 181cm，weight 85kg，no gait-disorder related diseases.
Measured repeatedly	Four groups of natural walking operation experiments were collected on the subjects.
Interval between four trials	0 week, 1 week, 3 weeks, 7 weeks
A group of natural walking test	The subjects walked normally 5 times effectively.
Effective normal walking	The subject maintains a natural state, and the walking distance includes more than 3 complete gait cycles.

Statistic calculation:

(1) A complete gait cycle collected in the natural walking test is normalized to 100%, and 0% is heel strike.
(2) The data of all gait periods obtained from 5 effective normal walks in a group of natural walking test were taken to obtain an average gait period (Select the left leg data uniformly).
(3) Calculate the ICC of the average gait cycle data obtained in different periods of the subjects to evaluate the reliability of repeated measures.
(4) Calculate the ROM of lower joints on each plane in the mean gait cycle.
(5) Calculate the RSD of the angular mobility in different walking phases and evaluate the precision of the test results.
(6) Calculate the RSD of the spatiotemporal parameters in different walking phases.

Results:

Test A

ICC of the average gait cycle data obtained by all different experimenters

Reliability coefficient	Flexion/Extension (X)	Abduction/Adduction (Y)	Internal/External rotation (Z)
Hip	0.990	0.993	0.915
Knee	0.997	0.977	0.909
Ankle	0.982	0.908	0.956

RSD of the angular mobility

RSD	Flexion/Extension (X)	Abduction/Adduction (Y)	Internal/External rotation (Z)
Hip	6.84%	2.56%	2.35%
Knee	2.27%	4.82%	7.70%
Ankle	3.18%	9.17%	5.16%

RSD of the spatiotemporal parameters

	Walking Speed (m/s)	Stride Width (m)	Stride Length (m)	Cycle time (s)
Spatiotemporal parameters	1.18±0.01	0.15±0.004	1.35±0.09	1.14±0.02
RSD	8.57%	2.78%	6.95%	1.60%

Test B

ICC of the average gait cycle data obtained in different gait periods of the subjects

Reliability coefficient	Flexion/Extension (X)	Abduction/Adduction (Y)	Internal/External rotation (Z)
Hip	0.977	0.985	0.960
Knee	0.951	0.932	0.918
Ankle	0.980	0.955	0.945

RSD of the ROM of lower joints in different walking phases

RSD	Flexion/Extension (X)	Abduction/Adduction (Y)	Internal/External rotation (Z)
Hip	4.07%	10.42%	6.07%
Knee	3.84%	9.80%	3.08%
Ankle	5.80%	10.33%	10.197%

RSD of the spatiotemporal parameters in different walking phases

	Walking Speed (m/s)	Stride Width (m)	Stride Length (m)	Cycle time (s)
Spatiotemporal parameters	1.36±0.04	0.12±0.012	1.38±0.04	1.04±0.03
RSD	2.85%	10.99%	3.08%	3.23%

Conclusion:

The results show that the ICC of the angular mobility of lower joints on three different planes obtained by all different experimenters (Test A) and the ICC of the average gait cycle data obtained in different gait phases (Test B) are all larger than 0.9, indicating that the device can be repeatedly measured between different experimenters and the gait parameters in different gait phases with high reliability, operation of the experimenter is qualified, the results obtained by the device and experimenter is highly reliable, and it can meet the requirements of this experiment.

It can also be found that the RSD of the angular mobility of the lower limbs in the gait cycle in both tests is less than 11%, and the RSD of the gait spatiotemporal parameters in both tests is less than 11%, indicating that the data obtained in two tests is pretty precise.
